# HIV-1 Nef Induces Proinflammatory State in Macrophages through Its Acidic Cluster Domain: Involvement of TNF Alpha Receptor Associated Factor 2

**DOI:** 10.1371/journal.pone.0022982

**Published:** 2011-08-23

**Authors:** Giorgio Mangino, Zulema A. Percario, Gianna Fiorucci, Gabriele Vaccari, Filippo Acconcia, Cristiano Chiarabelli, Stefano Leone, Alessia Noto, Florian A. Horenkamp, Santiago Manrique, Giovanna Romeo, Fabio Polticelli, Matthias Geyer, Elisabetta Affabris

**Affiliations:** 1 Department of Biology, University Roma Tre, Rome, Italy; 2 Department of Medical-Surgical Sciences and Biotechnologies, Sapienza University of Rome, Rome, Italy; 3 Institute of Molecular Biology and Pathology, Consiglio Nazionale delle Ricerche, Rome, Italy; 4 Department of Food Safety and Veterinary Public Health, Istituto Superiore di Sanità, Rome, Italy; 5 Abteilung Physikalische Biochemie, MPI für Molekulare Physiologie, Dortmund, Germany; Institut Pasteur Korea, Republic of Korea

## Abstract

**Background:**

HIV-1 Nef is a virulence factor that plays multiple roles during HIV replication. Recently, it has been described that Nef intersects the CD40 signalling in macrophages, leading to modification in the pattern of secreted factors that appear able to recruit, activate and render T lymphocytes susceptible to HIV infection. The engagement of CD40 by CD40L induces the activation of different signalling cascades that require the recruitment of specific tumor necrosis factor receptor-associated factors (*i.e.* TRAFs). We hypothesized that TRAFs might be involved in the rapid activation of NF-κB, MAPKs and IRF-3 that were previously described in Nef-treated macrophages to induce the synthesis and secretion of proinflammatory cytokines, chemokines and IFNβ to activate STAT1, -2 and -3.

**Methodology/Principal Findings:**

Searching for possible TRAF binding sites on Nef, we found a TRAF2 *consensus* binding site in the AQEEEE sequence encompassing the conserved four-glutamate acidic cluster. Here we show that all the signalling effects we observed in Nef treated macrophages depend on the integrity of the acidic cluster. In addition, Nef was able to interact *in vitro* with TRAF2, but not TRAF6, and this interaction involved the acidic cluster. Finally silencing experiments in THP-1 monocytic cells indicate that both TRAF2 and, surprisingly, TRAF6 are required for the Nef-induced tyrosine phosphorylation of STAT1 and STAT2.

**Conclusions:**

Results reported here revealed TRAF2 as a new possible cellular interactor of Nef and highlighted that in monocytes/macrophages this viral protein is able to manipulate both the TRAF/NF-κB and TRAF/IRF-3 signalling axes, thereby inducing the synthesis of proinflammatory cytokines and chemokines as well as IFNβ.

## Introduction

Several lentiviruses have evolved to infect non-dividing tissue macrophages. Some non-primate lentiviruses, such as caprine arthritis and encephalitis virus (CAEV) and Maedi-Visna virus exhibit a restricted tropism for monocytes/macrophages [1]–[3], whereas feline, simian and human immunodeficiency viruses (FIV, SIV and HIV, respectively) have acquired a wider tropism and an expanded range of target cells, involving CD4^+^ T lymphocytes beside macrophages [4]–[7]. The infection of CD4^+^ T cells accounts for many aspects of the viral pathogenesis, even if the capacity of lentiviruses to infect macrophages and other antigen-presenting cells (APC) plays a determinant role in the establishment, spreading and persistence of the infection. In case of HIV-1 infection, the selective sexual transmission of monocytotropic CCR5-restricted viruses has been reported [8], [9] indicating that macrophage tropism may facilitate establishment of virus infection in the newly infected host. The capacity of monocytes and macrophages to migrate in organs and to survive in tissues makes them potential conveyors of HIV-1 infection. Moreover, unlike T cells, HIV-infected macrophages appear to be resistant to the cytopathic effects of the virus and to the current antiretroviral therapies [10], thus serving as a reservoir for persistent infection.

Lentiviruses, in general, and HIV-1, in particular, impair macrophage functions and alter the cytokines and chemokines production pattern, resulting in chronic infection, tissue damage and recruitment of target cells in the site of primary infection. It has been demonstrated that in infected macrophages Nef mediates lymphocyte chemotaxis and activation through the synthesis and the release of MIP-1α, MIP-1β and other soluble factors, thus improving lymphocyte recruitment and activation at virus replication sites [11], [12]. It has also been shown that Nef is able to regulate the expression of some inflammatory cytokines such as IL-1β, IL-6, TNF-α and the production of IFNβ in infected cells as well as in uninfected macrophages treated with the viral protein [13], [14].

HIV-1 Nef is a small myristoylated, cytoplasmic, multifunctional protein (MW 27–34 kDa) that acts as an adaptor molecule partially associated with the cell membrane [15]. Nef-defective viruses lead to an attenuated clinical phenotype with reduced viral load in mouse models, monkeys, and human disease [16]–[20]. In addition, *nef* transgenic mice develop an AIDS-like disease [21] suggesting a pathogenic role for this HIV-1 accessory factor. Several functions are ascribed to the interaction between Nef and cellular counterparts; some of them, such as CD4 and class I MHC downregulation, are detected in both T lymphocytes and monocytes/macrophages, whereas others are cell type specific. In CD4^+^ T cells Nef interacts with TCR chain, Lck and PAK kinases and the molecular adapters LAT and Vav thereby inducing a pre-activation state in infected T cells. On the other hand, the molecular mechanisms that allow the activation of monocytes/macrophages and the synthesis and release of inflammatory cytokines are less well characterized, even if we and others reported that in macrophages Nef is able to activate NF-κB signalling pathways and that this activation is responsible for the production of cytokines and for the increased infection of resting T lymphocytes [12], [14].

Here we report that signalling events required for the synthesis and the release of inflammatory factors and IFNβ in human monocytic cells treated with myristoylated recombinant Nef require the integrity of the so called Nef acidic cluster (AC) made by a four-glutamate stretch located in the N-terminus arm of the protein. Modelling analyses reveal that AQEEEE sequence perfectly matches the *consensus* binding sequence for the TRAF2 adapter protein and it is also compatible with the binding to TRAF6. Pull-down assays demonstrate the ability of Nef to form complexes only with TRAF2, whereas specific RNA silencing suggests the involvement of both TRAF2 and TRAF6.

## Results

### The Nef Acidic Cluster is a putative TRAF2 consensus binding site

**Figure 1:**
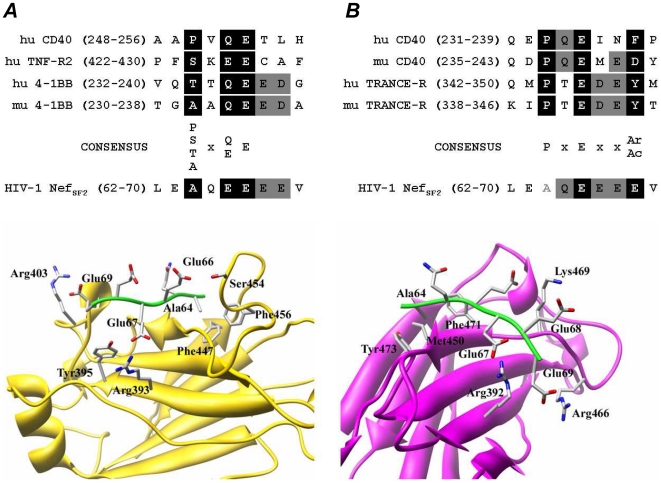
Putative complexes formed by Nef Acidic Cluster with TRAF2 and TRAF6. Upper panels: sequence alignment between Nef AC and TRAF2 (**A**) or TRAF6 (**B**) binding sites in known TRAF interactors. The conserved residues within the *consensus* sequence are in black background whereas the residues present in Nef AC as well as in TRAF2 and TRAF6 interactors are in gray background. Mismatched Ala in Nef compared to TRAF6 *consensus* in **(B)** is in gray. TRAFs interactors sequences are as in [22], [23]. Lower panels: schematic representation of the modelled complexes formed by Nef AC with TRAF2 (**A**) and TRAF6 (**B**). Nef AC-TRAF2 complex has been modelled using the three-dimensional structure of the TRAF2/4-1BB complex (PDB code: 1D0J) [22] as a template. Nef AC backbone is shown in green, TRAF2 backbone in gold. Nef AC-TRAF6 complex has been modelled using the three-dimensional structure of the TRAF6/RANK complex (PDB code 1LB5) [23]. Nef AC backbone is shown in green, TRAF6 backbone in magenta.

**Figure 2:**
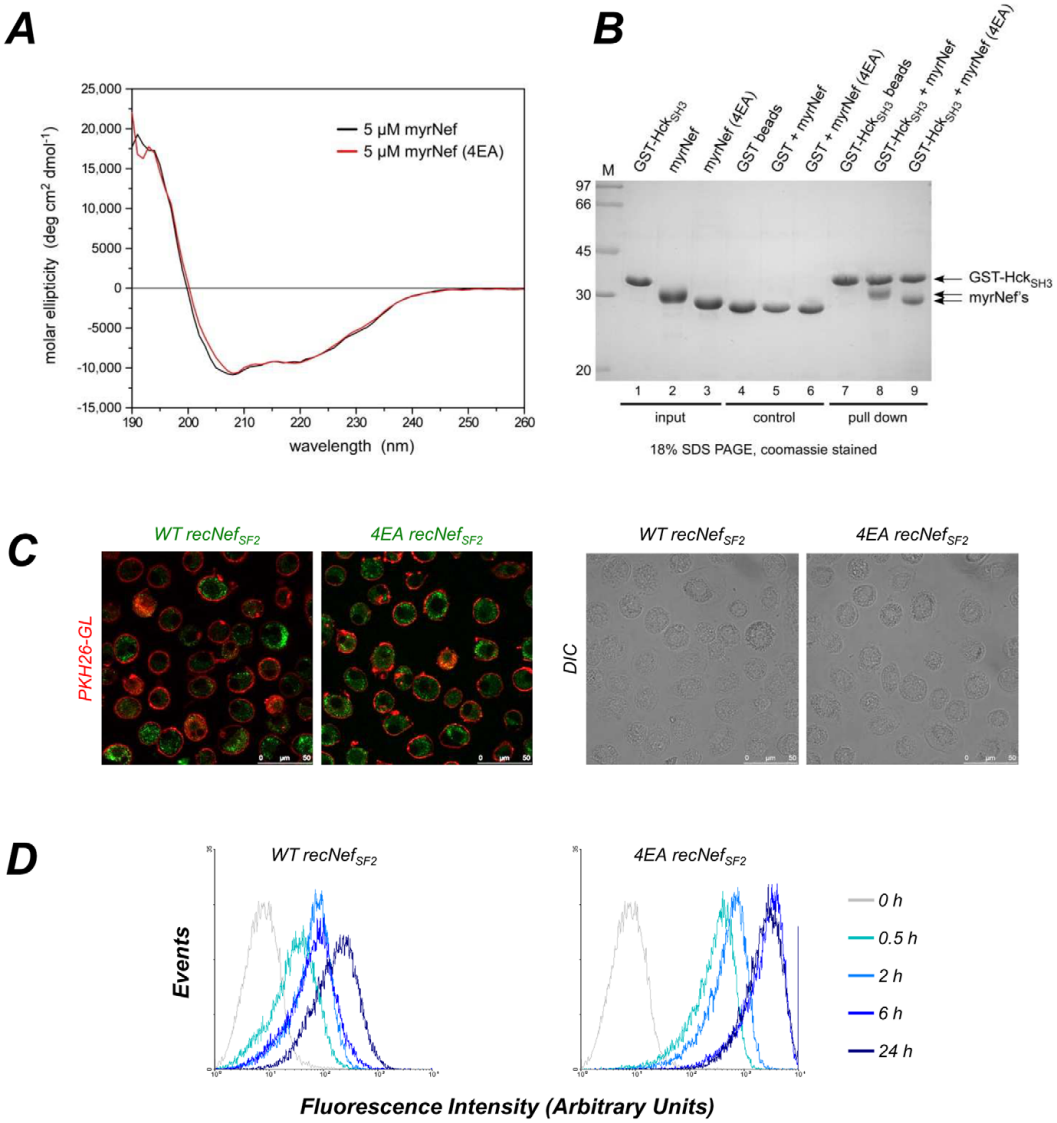
Acidic Cluster Nef mutant characterization. (**A**) Circular dichroism spectroscopy analysis of myr^+^ wild type recNef protein (black line) and myr^+^ 4EA recNef mutant (red line) revealed similar secondary structure content from both proteins. (**B**) GST-HCK_SH3_ pull down assays with recombinant Nef protein showed binding to both wild type Nef and the 4EA mutant in a comparable manner (lanes 7–9), supporting the structural integrity of the protein. For input control, recNef proteins were similarly incubated alone (lanes 1–3) or in presence of GST beads (lanes 4–6). (**C, D**) Wild type Nef and 4EA mutants are internalized by MDMs. Cells were treated with 300 ng/ml of the corresponding recombinant proteins labelled with AlexaFluor488 green dye. For CM (**C**), MDMs were cultured on glass bottom dishes and treated for 30′ wt or 4EA myr^+^ recNefSF2-Alexa488, then counterstained with PKH26 red fluorescent dye, fixed and analysed. Left panels: merged images of stained cells. Right panels: Differential Interference Contrast (DIC) images. (**D**) For CFM analysis cells were treated for the indicated times with wild type (left panel) or 4EA (right panel) recNef proteins. Gray plot: untreated cells; sky blue: 30′: light blue: 2 h; blue: 6 h; dark blue 24 h.

The comparison between HIV-1 Nef primary structure and TRAF2 *consensus* binding sequence [22] reveals the presence of a putative binding motif in the sequence A^64^QEEEE^69^ (numeration according to SF2 HIV-1 allele) encompassing the Nef AC (Fig. 1A). Modelling analysis performed using crystallographic data obtained on TRAF2/4-1BB complex [22] shows that the stereochemistry of Nef 64–69 sequence stretch is highly compatible with TRAF2 binding site. In particular, in the modelled complex, Nef Ala^64^ is positioned inside a hydrophobic cavity lined by TRAF2 residues Phe^447^ and Phe^456^, Nef Glu^66^ forms a hydrogen bond with TRAF2 Ser^454^, and Nef Glu^67^ and Glu^69^ form salt bridges with TRAF2 Arg^393^ and Arg^403^, respectively. On the other hand, the comparison between the reported TRAF6 *consensus* binding sequence [23] and the A^64^QEEEE^69^ motif indicates that the two sequences are superimposable except for Ala^64^ (Fig. 1B). Nevertheless, modelling analysis performed using the crystallographic data obtained on TRAF6/RANK complex [23] reveals that, even if the interaction of Nef Ala^64^ with TRAF6 Phe^471^, Met^450^ and Tyr^473^ residues is probably weaker than the interactions made by the Pro residue of the *consensus* sequence, electrostatic interactions involving Glu^67^, Glu^68^ and Glu^69^ of Nef with Arg^392^, Lys^469^ and Arg^466^ of TRAF6, respectively, can stabilize the interaction between Nef and TRAF6 (Fig. 1B). Therefore, we produced myristoylated recombinant Nef_SF2_ (myr^+^ recNef) by substituting E^66^EEE^69^ with four alanines (mutant 4EA) testing if AC mutation impact the overall Nef structure. As reported in Fig. 2A, both myristoylated wild type Nef and the 4EA mutant displayed similar profiles in circular dichroism analysis, supporting that the overall structure is maintained in the mutant protein. In addition, both proteins equally well interacted with the Hck-SH3 domain, a well conserved function mediated by the Nef proline rich region [24] (Fig. 2B). We also assessed the ability of AC-mutated recNef to enter MDMs. To this purpose, myr^+^ recNef 4EA as well as myr^+^ wt proteins were AlexaFluor 488 labelled. MDMs were, then, incubated with the fluorescent proteins and analyzed by confocal microscopy and cytofluorimetry. Confocal microscopy analysis showed a superimposable punctuate intracytoplasmic pattern of wt and 4EA mutant (Fig. 2C). Cytofluorimetric analysis revealed that not only myr^+^ 4EA recNef is internalized as the wt protein, but it seems to be internalized even with a faster rate (Fig. 2D). As negative controls we observed that recNef internalization was abrogated at +4°C and that both a signalling inactive Nef mutant (i.e. G2A, see [13] and later in this article) as well as an unrelated protein (Ovalbumin-FITC) were internalized, but were unable to induce STAT1 and STAT2 tyrosine phosphorylation (data not shown). Collectively, these results indicate that the EEEE→AAAA mutation impairs neither the overall structure nor the ability of the protein to be internalized by MDMs.

### Nef AC is essential to induce NF-κB in MDMs

**Figure 3:**
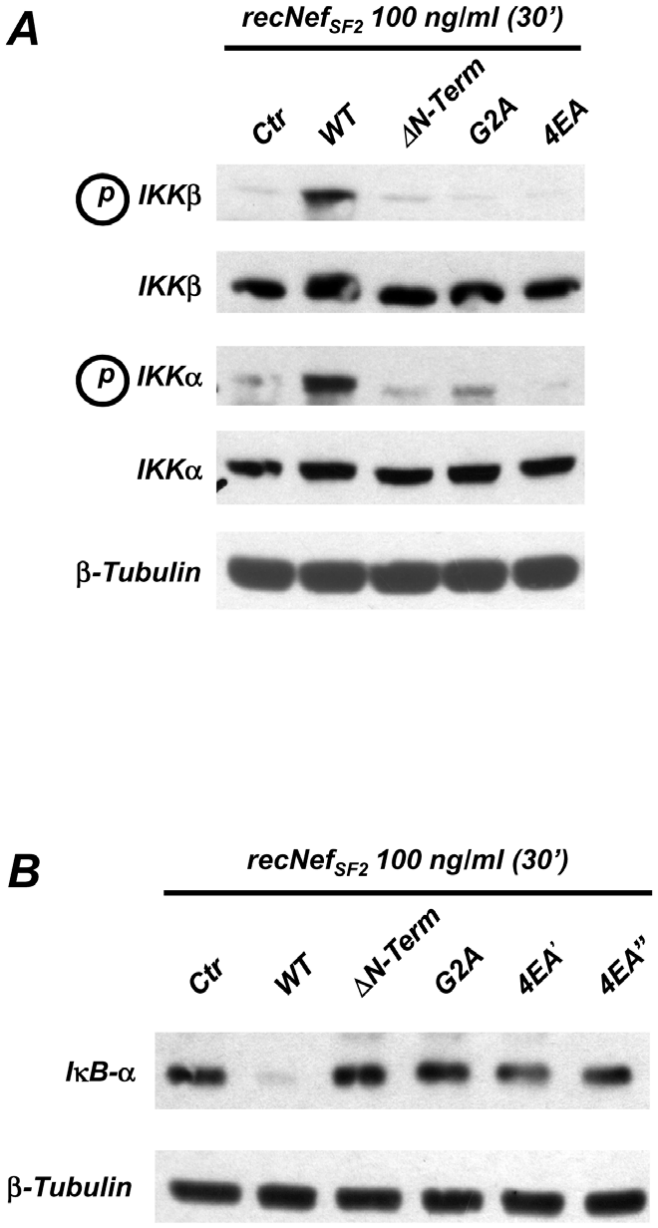
Nef induces NF-κB signalling in an Acidic Cluster dependent manner. Seven days old human primary MDMs were treated for 30′ with 100 ng/ml of myr^+^ wild type (WT) recNef, the protein lacking the first 44 aminoacids (ΔN-Term), mutated in the myristoylation site (G2A) or myr^+^ recNef 4EA mutant. Total cell protein extracts (50 µg) were analyzed by Western Blot as reported in Materials and Methods section using specific anti phospho-IKKα/IKKβ and anti IKKα/IKKβ (**A**) or with anti IκB-α (**B**) antibodies. In both (**A**) and (**B**) β-tubulin steady-steate expression level was used as an internal loading control. Results shown represent one out three different donors.

Based on the structural models as well as on previous results showing that Nef is able to intersect the CD40 signalling [12] and to trigger NF-κB, MAPKs and IRF-3 signalling pathways [13], we evaluated the possible involvement of the AC domain in NF-κB pathway. Both myr^+^ wild type and 4EA recNef proteins were used to stimulate primary human MDMs. As negative controls we used recNef mutants lacking the myristoylation site (G2A) or the first 44 amino acids (N-term.) as we previously demonstrated that both did not activate NF-κB, MAPKs and IRF-3 in treated MDMs [13]. NF-κB activation was evaluated by testing both serine phosphorylation of the catalytic subunits of the IKK complex (namely IKKα and IKKβ) as well as degradation of the inhibitory factor IκB-α. A 30′ treatment of MDMs with myr^+^ wt recNef induced the phosphorylation of both IKKα and IKKβ (Fig. 3A) and degradation of IκB-α (Fig. 3B), whereas myr^+^ 4EA recNef was unable to induce both the activation of IKKs and the degradation of IκB-α. The lack of IκB-α degradation was observed using two different batches of the 4EA mutant (*i.e.* myr^+^ recNef 4EA′ and 4EA″, Fig. 3B). These results demonstrate that both Nef myristoylation and the AC are necessary for the activation of NF-κB.

### Proinflammatory cytokine induction requires the integrity of Nef AC

**Figure 4:**
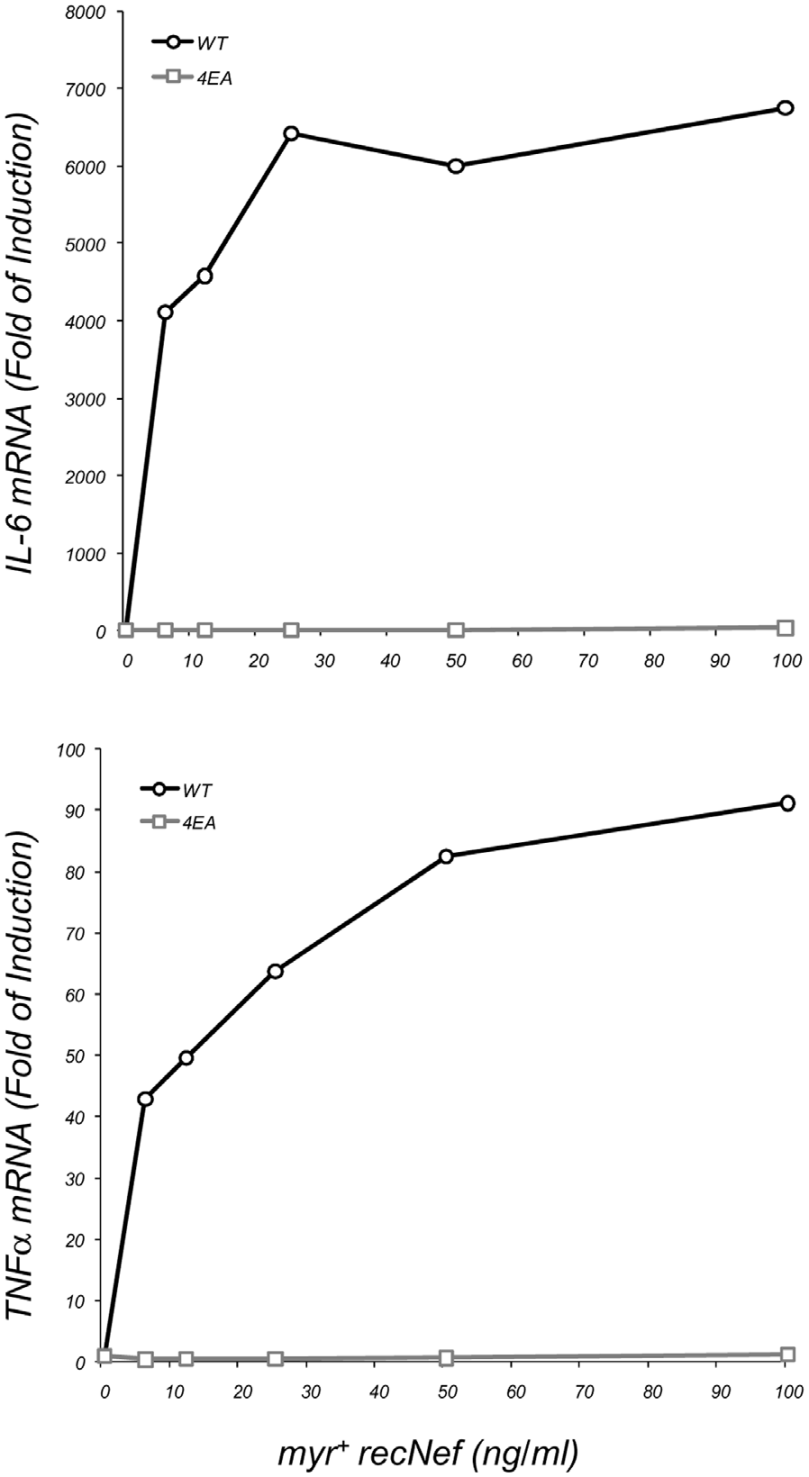
Dose-response analysis of IL-6 and TNFα mRNA. Primary human MDMs were treated for 2 h with the indicted doses of myr^+^ wild type recNef (dark line, empty circles) or myr^+^ 4EA recNef mutant (gray line, empty squares). Cells were processed as reported in the Materials and Methods section. Upper panel: IL-6 mRNA levels; lower panel: TNFα mRNA levels. Results in figure and in Table I represent two out of four independent experiments.

Myr^+^ recNef treatment of primary MDMs as well as infection of MDMs with *nef* espressing HIV-1 pseudotypes induces the synthesis and the release of several inflammatory cytokines and chemokines (*i.e.* IL-1β, IL-6, TNFα, MIP-1α and MIP-1β) [11], [14]. The expression of all these factors is tightly regulated by the presence in their promoter sequences of κB responsive elements [25]–[28]. Therefore, we checked if myr^+^ 4EA recNef was able to induce the synthesis of these cyto- and chemokines in MDMs as we already reported for wt recNef [14]. Human MDMs were treated for 2 h with 100 ng/ml of myr^+^ wt recNef or with G2A, ΔN-term and myr^+^ 4EA recNef. Total mRNA was analyzed by RT-PCR using specific primers for IL-1β, IL-6, TNFα, MIP-1α and MIP-1β. Results in Table 1 show a marked inhibition of all these proinflammatory cytokines and chemokines transcripts (ranging from 82% for CCL4/MIP-1β to up to 99% for IL-6) using two different preparations of the myr^+^ 4EA recNef. Dose-response analysis reveal that as low as 6.25 ng/ml of myr^+^ wild type recNef was able to induce both TNFα and IL-6 mRNAs whereas 4EA mutant was unable to induce these mRNAs at all doses tested (Fig. 4). Using the IKKs specific inhibitor BMS-345541 [29] we also demonstrated that the Nef-induced TNFα and IL6 mRNA upregulation is dependent on NF-κB activation (Figure S1).

**Table 1:** RT-PCR analysis of cytokines and chemokines induced in MDMs following recNef treatment.

MDM Treatment (2 h)	n-Fold Induction* (% of Inhibition**)				
	IL-1β	IL-6	TNF-α	CCL3/MIP-1α	CCL4/MIP-1β
None	1	1	1	1	1
wt recNef_SF2_	357.05	7486.11	249.00	324.03	303.38
G2A recNef_SF2_	1.80 (99.49)	6.92 (99.9)	3.31 (98.67)	1.77 (99.45)	9.13 (96.99)
ΔN-Term recNef_SF2_	9.92 (97.22)	83.58 (98.88)	11.20 (95.5)	6.70 (97.93)	18.96 (93.75)
4EA′ recNef_SF2_	6.82 (98.09)	39.95 (99.46)	9.06 (96.36)	6.23 (98.08)	16.11 (94.69)
4EA″ recNef_SF2_	36.50 (89.78)	487.75 (93.48)	27.57 (88,93)	23.18 (92.85)	52.71 (82.62)

*Expressed using as reference the value obtained in untreated cells.

**Calculated considering as reference the value obtained in MDMs treated with wt Nef.

### IRF-3 activation and IFNβ production is dependent on Nef AC

**Figure 5:**
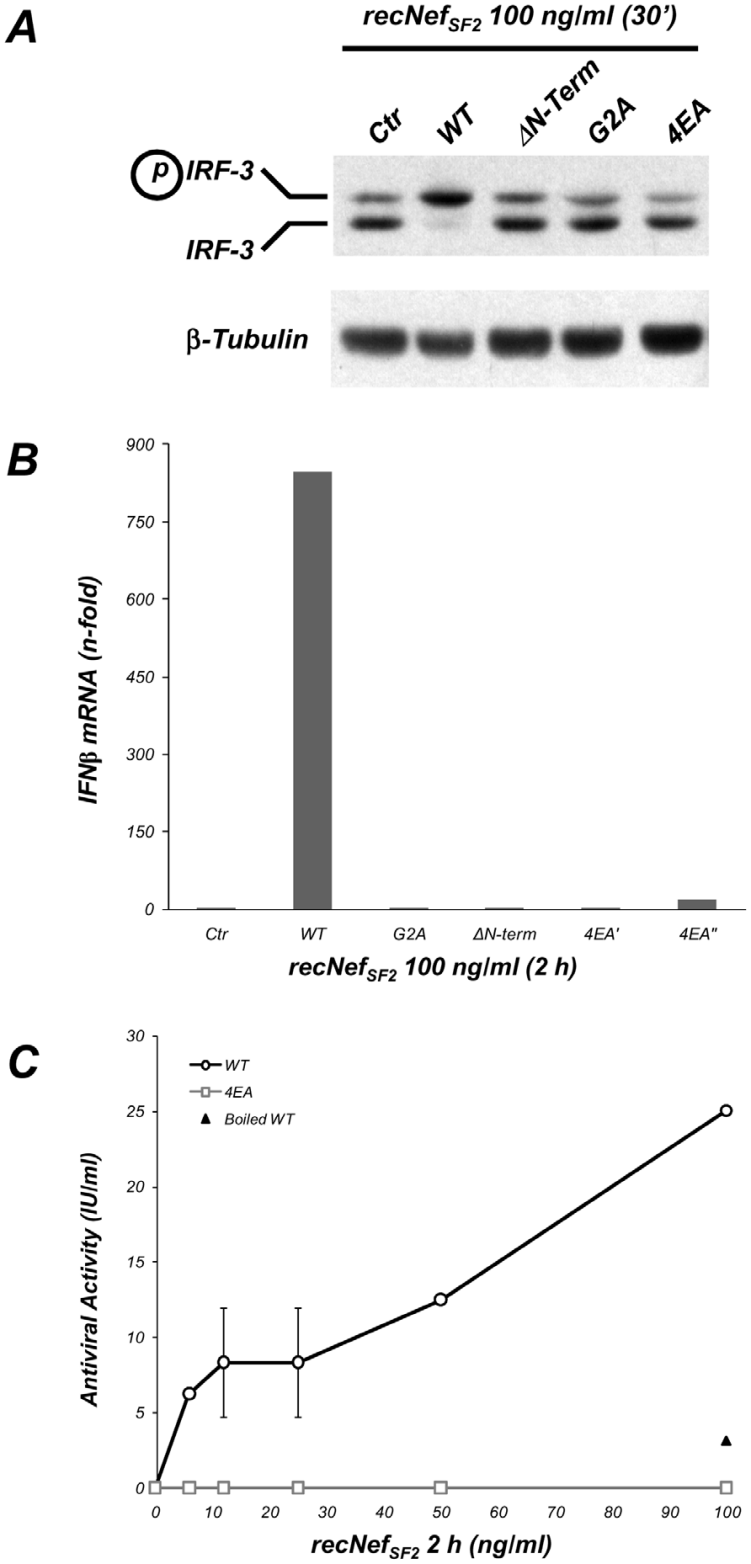
Nef-dependent activation of IRF-3 and synthesis of IFNβ requires the integrity of the Acidic Cluster. (**A**) MDMs were treated with myr^+^ wt as well as with ΔN-Term, G2A or myr^+^ 4EA recNef (100 ng/ml for 30′). Total cell protein extracts (30 µg) were analyzed by Western Blot using anti-IRF3 specific antibodies. IRF-3 activation was visualized as accumulation of the slower migrating form of the protein corresponding to the iperphosphorylated form and the decrease of the faster migrating band. β-tubulin steady-steate expression level was used as an internal loading control. (**B**) MDMs were treated for 2 h with myr^+^ wt as well as with ΔN-Term, G2A or myr^+^ 4EA recNef (100 ng/ml, 2 h). Cell were processed, total RNA was isolated and Real Time PCR was performed as described in Material and Methods section. Results are expressed as fold of induction using the levels of IFNβ mRNA expression in untreated cells as reference. (**C**) Supernatants collected from MDMs treated for 2 h with twofold dilution of myr^+^ wt (black line, empty circles), 4EA (gray line, empty squares) or 100 ng/ml of heat-inactivated myr^+^ wt recNef (black triangle), were tested for the induction of the antiviral state on A549 indicator cells. Results are expressed as International Units (IU) of IFN/ ml. Results were obtained from four independent healthy donors.

We previously reported that the myr^+^ recNef treatment of primary human MDMs induces the activation of the IRF-3 transcription factor that in turn allows the synthesis and release of IFNβ [13]. Several receptors induce type I IFNs using as signalling intermediates TRAF family members *e.g.* the TLR4 and TLR3 members of the Toll Like Receptor family [30]–[32]. Therefore, we supposed that the putative TRAF2 and/or TRAF6 binding motif could be involved also in the Nef-induced IRF-3 activation and in the IFNβ production. To test this hypothesis primary MDMs were treated with myr^+^ wt as well as with myr^+^ 4EA recNef, evaluating the phosphorylation status IRF-3 as shift in the electrophoretic migration of the protein. As shown in Fig. 5A, treatment of MDMs with the wt protein induced the accumulation of a slower migrating hyperphosphorylated form of IRF-3 with the concomitant disappearance of the faster migrating one. Conversely, treatment with the myr^+^ 4EA, the G2A, and the ΔN-term protein was unable to activate IRF-3. We also tested the induction of the mRNA coding for IFNβ and the release of this cytokine in the supernatant of Nef-treated MDMs, testing the antiviral activity. Again, myr^+^ 4EA recNef did not upregulate IFNβ mRNA (Fig 5B) and failed the release of IFNβ at all doses we tested (Fig. 5C). A dose as low as 6.25 ng/ml of myr^+^ wild type recNef induced the release of IFNβ. These results demonstrate that the AC is required also for synthesis and release of IFNβ.

### Both myristoylation and AC are essential to induce the tyrosine phosphorylation of STAT1, STAT2 and STAT3

**Figure 6:**
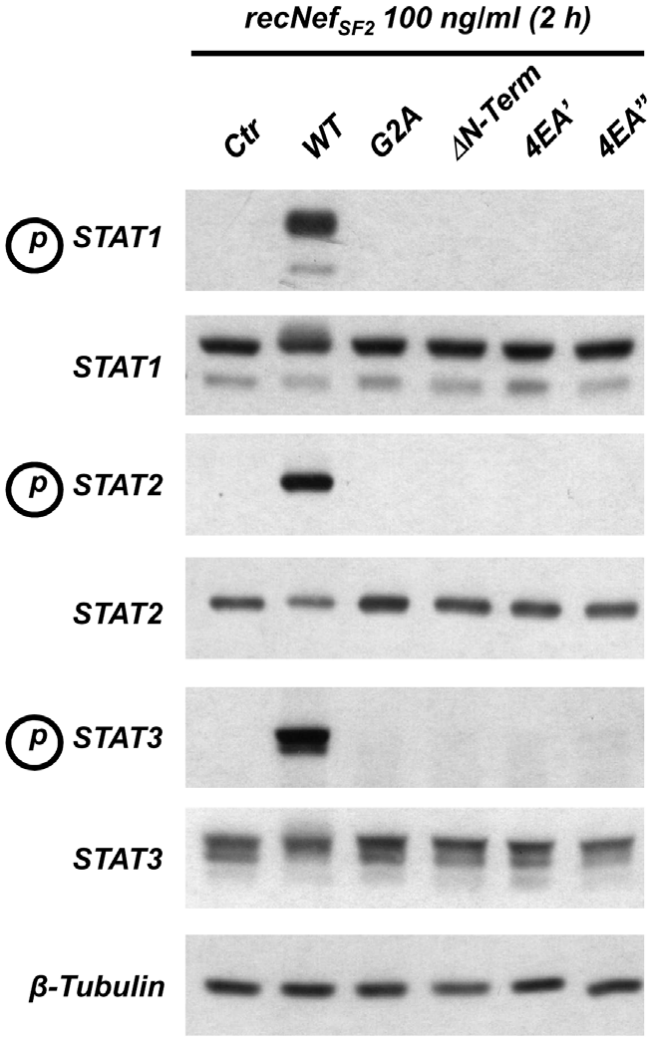
Tyrosine phosphorylation of STAT1, STAT2 and STAT3 requires the Nef Acidic Cluster. MDMs were treated for 2 h with 100 ng/ml of myr^+^ wt as well as with ΔN-Term, G2A or myr^+^ 4EA recNef. Total cell protein extracts (50 µg) were analyzed by Western Blot using specific anti phosphotyrosine-STAT1, anti phosphotyrosine-STAT2 and anti phosphotyrosine-STAT3 antibodies. Steady-state expression levels of STAT1, STAT2 and STAT3 were evaluated using corresponding specific antibodies whereas β-tubulin steady-steate expression level was used as an internal loading control. Results were obtained from four independent healthy donors.

The release of the inflammatory cyto- and chemokines and IFNβ induces in MDMs a second wave of activation through the JAK/STAT signalling pathway. In particular, tyrosine phosphorylation of STAT1, STAT2 and STAT3 was observed [13], [33], [34]. Based on the above-reported results it is plausible to suppose that myr^+^ 4EA recNef was unable to induce the activation of these signal transducers and activator of transcription. As expected, myr^+^ 4EA, G2A, ΔN-term recNef were unable to induce the tyrosine phosphorylation of STAT1, -2 and -3 in primary MDMs (Fig. 6). Again, we observed the lack of STATs tyrosine phosphorylation using two different 4EA mutant preparations. These results indicate that the E^66^EEE^69^ motif is also essential for the induction of this second JAK/STAT-based wave of regulation.

### The AC mutation markedly reduces *in vitro* interaction of wt Nef with TRAF2

**Figure 7:**
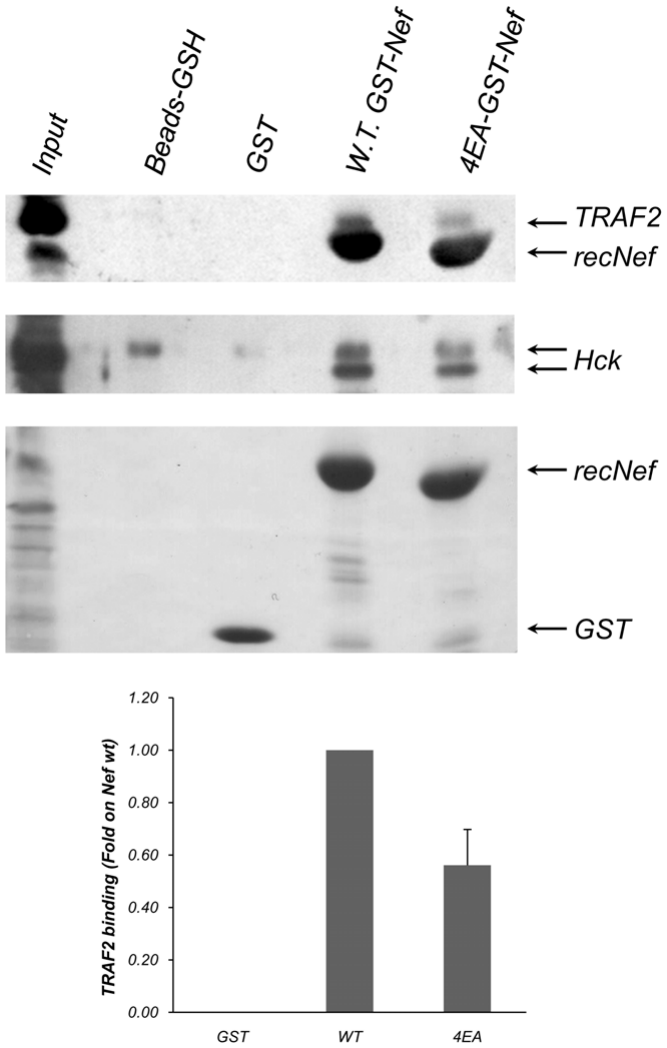
Nef interacts *in vitro* with TRAF2. Recombinant GST-Nef proteins (WT or 4EA mutants) were incubated with THP-1 total cellular lysates. Pull-down assay was performed as reported in Materials and Methods section and samples were analyzed on 9% SDS-PAGE. Western Blot was performed using anti-TRAF2 (Upper panel) or anti-Hck (Middle panel) specific antibodies. As gel loading control, samples were analyzed on SDS-PAGE and proteins were detected by Ponceau S staining (Lower panel). Band intensities were quantified using ImageJ v1.32J and data were normalizing using Hck signals as calibrator. Mean intensity as well as standard deviation was calculated using data collected from three independent experiments and statistical analysis performed using InStat3 and ANOVA test.

As reported in the first paragraph and in Fig. 1, Nef AC is part of a *consensus* binding sequence for TRAF2 and fits with the TRAF6 binding surface. To test if Nef and TRAFs could physically interact *in vitro*, both wt and 4EA Nef-GST fusion proteins were produced and used in pull down assays. As depicted in Fig. 7, wt Nef-GST was able to pull down TRAF2, whereas a statistically significant reduction in this interaction was detectable using the 4EA Nef-GST preparation (p>0.05 using ANOVA test). The 4EA mutant still retain the ability to bind Hck at comparable levels to wt Nef (Fig. 7, middle panel and Fig. 2B), thereby demonstrating that the AC mutation does not affect the overall Nef binding ability with other known interactors. As negative control, GST alone was unable to bind any TRAF2. Neither wt nor 4EA Nef-GSTs were able to pull down TRAF6 (data not shown) indicating that, at least *in vitro*, this interaction was not detectable. We also tried to perform *in vivo* Nef-TRAF2 and Nef-TRAF6 co-immunoprecipitation assay on Nef-treated MDMs in a time course experiment (5′ to 30′) but we didn't record any signals because the amount of recNef internalized by MDMs is probably too low to allow detection by Western Blot (data not shown).

### Silencing of both TRAF2 and TRAF6 inhibits the Nef-induced STATs tyrosine phosphorylation

**Figure 8:**
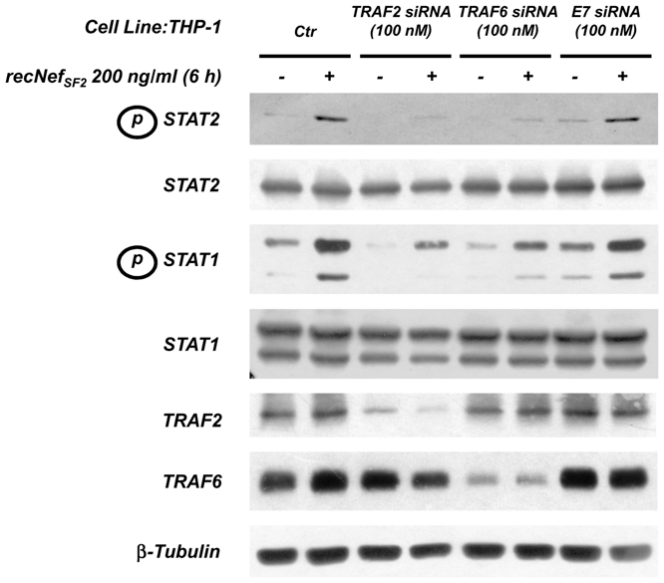
Both TRAF2 and TRAF6 silencing interfere with Nef-induced STAT1 and STAT2 tyrosine phosphorylation. THP-1 cells were transfected with specific TRAF2, TRAF6 or unrelated (*i.e.* for HPV16 E7) siRNA as described in Material and Methods section. Cells were then incubated with recNef (200 ng/ml) for 6 h. Total cell protein extracts (50 µg) were analyzed by 7% SDS-PAGE and Western Blot was performed using specific anti phosphotyrosine-STAT1 and anti phosphotyrosine-STAT2 antibodies. STAT1 and STAT2 steady-state expression levels were checked using the corresponding specific antibodies. Anti-TRAF2 and anti-TRAF6 antibodies were used to verify the specificity of silencing, ruling out off-target effects. β-tubulin steady-state expression level was used as an internal loading control. Results reported in figure represent one out of three independent silencing experiments.

The above described experiments didn't formally demonstrate the involvement of TRAF family members in signalling effects induced by Nef in MDMs. To confirm the involvement of TRAF2 and/or TRAF6 in these events, silencing experiments were performed on the human monocytic cell line THP-1. Indeed, recNef treatment induces in these cells signalling effects superimposable to those observed in primary human MDMs (G. Mangino, unpublished data). THP-1 cells were transfected with TRAF2 or TRAF6 specific siRNA pools. Seventy-two hours later, cells were treated with myr^+^ wt recNef for 6 h. As shown in Fig. 8, recNef induced only a very light STAT1 and STAT2 tyrosine phosphorylation signal in TRAF2 silenced cultures. Surprisingly enough, a similar result was obtained even in TRAF6 silenced cultures. As a negative control, THP-1 cells silenced with unrelated siRNA (*i.e.* HPV16 E7 siRNA) responded to myr^+^ recNef treatment as well as control cultures. Therefore both TRAF2 and TRAF6 appear to be involved in the cytokines-mediated activation of STAT1 and STAT2.

## Discussion

Emerging evidences point out the ability of the HIV-1 Nef protein to hijack cellular signalling pathways to promote viral replication and spreading. Nef affects the function of macrophages inducing the production of two CC-chemokines, (*i.e.* CCL3/MIP-1 and CCL4/MIP-1β) possibly to recruit CD4^+^ T cells at the sites of virus replication, and induces the release of soluble factors from macrophages (*i.e.* sICAM-1 and sCD23) stimulating B cells to render resting T lymphocytes more permissive to HIV-1 infection and replication [11], [12]. These findings suggest that Nef influences the activation status of the infected cells to generate a suitable environment for virus production also increasing the susceptibility of the surrounding cellular reservoir to infection. Recent observations also indicate a role of Nef to interfere with signalling events in uninfected cells that are able to pick it up from the extracellular milieu or by cell to cell transfer [35]–[37].We have already reported that exogenously added Nef induces on *in vitro* cultured MDMs the synthesis and the release of proinflammatory cytokines and chemokines and IFNβ in a NF-κB dependent manner. After 1.5/2 h of cell treatments with the viral protein the pool of secreted factors are able to activate STAT1, STAT2 and STAT3 in an autocrine and paracrine manner [13], [14], [33], [34]. Nevertheless the molecular mechanisms involved in these phenomena as well as the Nef structural domains involved in these activations remained elusive. Results reported here clearly indicate that beside the myristoylation site, already identified as an essential hallmark to achieve NF-κB, IRF-3 and STATs activations, the other main structural domain involved in these functions is the N-terminal acidic cluster E^66^EEE^69^. Fig. 3 shows that Glu to Ala substitutions within the AC in the myr^+^ 4EA mutant completely abrogate the ability of Nef to induce IKKs phosphorylation and IκB degradation, thereby preventing the activation of NF-κB signalling events. Due to the strict interdependence between the activation of the canonical NF-κB pathway and all the downstream events, the overall effect of this Nef mutation is the lack of induction of proinflammatory state, attested by the unsuccessful induction of specific cytokines and chemokines mRNA, as well as the lack of the subsequent STATs tyrosine phosphorylation (see Table 1 and Figs. 4 and 6). In addition, results shown in Fig. 5A demonstrate that the 4EA mutation, affecting the Nef-induced IRF-3 phosphorylation, also prevents the synthesis and the release of IFNβ (Fig. 5B and 5C). We obtain these results by treating cells with 100 ng/ml recNef except for confocal microscopy analysis and silencing experiments in which 300 and 200 ng/ml respectively were used. In particular, in the silencing experiments we used THP-1 cells that are not differentiated macrophages and display a reduced uptake ability compared to MDMs. The used quantities are 10 to 30-fold higher to the amount of Nef found in the sera of HIV-1 infected individuals [38]. Nevertheless it has been speculated that this concentration might be even higher in the lymphonodal germinal centers, where virion-trapping dendritic cells, virion-infected CD4^+^ T cells and macrophages are densely packed [39]. In addition, the results shown in Figs. 4 and 5C demonstrate the induction of pro-inflammatory cytokines and IFNβ at doses as low as 6,25 ng/ml close to those recorded in the patients sera. These events require NF-κB activation and are mandatory for the subsequent STATs tyrosine phosphorylation.

Previous studies identified the AC as the domain mainly involved in the Nef-dependent downregulation of HLA-A and -B alleles [40]. Analyses performed on primary Nef structure evidenced the great conservation of this domain along Nef sequences derived from different HIV-1 laboratory adapted strains as well as primary isolates [41], [42]. In addition, we found a TRAF2 *consensus* binding site in the AQEEEE sequence encompassing the conserved four-glutamate acidic cluster. Even if some divergences from the TRAF2 *consensus* binding sequence were reported, it is noteworthy that they never affect the Glu^69^. Indeed, only a conservative Glu to Asp substitution was found in 2 out of 50 sequences recorded. In addition, just a Glu to Ser substitution was reported for Glu^67^ beside some conservative Glu to Asp substitutions (*i.e.* 15 out of 50 sequences) [42]. Glu^66^ was found to be substituted by Gln in 4 out of 50 sequences, thereby preserving the TRAF2 *consensus* sequence, whereas only a Glu^66^→Lys substitution was reported in a rapid progressor patient [42]. Further, just 1 out of 50 sequences showed a substitution in the Ala^64^ (*i.e.* from Ala to Arg) and this mutation characterizes the isolate from a non-progressor patient [42]. The structural models that we elaborated suggest that both Glu^67^ and Glu^69^ represent, indeed, key residues for the interaction with both TRAF2 and TRAF6 within the AC. Recently, Baugh and colleagues have reported that mutants containing one or both Glu^67^ and Glu^69^ mutated to Ala still retain the ability to downregulate HLA-A and -B alleles as well as to induce the activation of Pak-2 serine kinase [43]. These results indicate that the selective pressure imposing the conservation of an acidic residue in these two positions is not based on these functions, but it rather resides in other functions mediated by Nef. TRAFs silencing experiments (Fig. 8) suggest that the ability of the viral protein to activate the synthesis and the release of proinflammatory factors and IFNβ through TRAF2 and TRAF6 could be one of these functions.

As mentioned above, Swingler and colleagues describe that HIV-1 Nef intersects CD40 signalling in monocytes/macrophages thereby promoting the recruitment and infection of resting T cells [12]. It has been shown that CD40 transduces its activation signals engaging at least three different TNF receptor associated factors (*i.e.* TRAF2, -3 and -6) [44]–[48]. It has also been reported that TRAF2 is able to recruit the IKK complex through a direct interaction with the leucine zipper motifs of both IKK and IKKβ [49]. Pull down assay (Fig. 7) and TRAF2 RNA interference experiments (Fig. 8) suggest that Nef intersect CD40 signalling by a direct interaction with TRAF2 and subsequent recruitment and activation of IKK complex. The results reported here underline that this interaction involves the Nef AC. It is interesting to note that all the members of the TNF receptor family, including CD40, require trimerization to start signalling events and that Nef has also the tendency to make both dimers and trimers but not higher oligomers [50], opening the possibility that Nef needs to trimerize to allow TRAF2 recruitment and subsequent NF-κB activation. In our hands Nef was unable to interact *in vitro* with TRAF6 in pull down experiments (data not shown). Nevertheless, silencing experiments using specific TRAF6 siRNA indicate that also this adapter could be involved in the signalling events leading to the synthesis and the release of STAT1 and -2 activating factors. CD40 recruits TRAF6 through a direct interaction, thereby initiating a signalling cascade leading to activation of NF-κB and MAPKs [44], [51], whereas other receptors attract it through other adaptors such as MyD88 or IRAK-1 [52], [53]. The lack of any TRAF6 specific signal in pull down assays that we performed using Nef-GST fusion proteins could be due either to an indirect interaction between Nef and TRAF6 mediated by other molecules or to a weak direct interaction between these proteins that could not be detected by pull-down techniques.

It is noteworthy that other viruses evolved the ability to interact and/or interfere with TRAFs functions through a particular viral product. This is, indeed, the case of EBV LMP-1, HHV-8 K15, herpesvirus Saimiri STP-A and HCV NS5A [54]–[57]. We propose that Nef belongs to such a family of “viral hijackers” thus leading to the induction of the inflammatory state in MDMs by subduing TRAFs mediated signalling events to viral replication purposes.

## Materials and Methods

### Cells, recombinant Nef preparations and reagents

Peripheral Blood Mononuclear Cells (PBMCs) were isolated from buffy coats obtained from healthy donors at Centro Trasfusionale-Cattedra di Ematologia, Università degli Studi “La Sapienza” Rome. No ethical approval from our and university “La Sapienza” ethics committees nor formal or verbal informed consent from blood donors were necessary to use buffy coats as sources of primary monocytes. Cells were isolated by positive selection using CD14 microbeads and LS columns, all purchased from Miltenyi Biotech (Auburn, CA), following the manufacturer's recommendations. The purity of the recovered cell populations was assayed by cytofluorimetric analysis by means of phycoerythrin-conjugated anti-CD14 monoclonal antibody (clone UCHM-1, Chemicon-Cymbus, Temecula, CA) labelling. Cell preparations staining below 95% positive for CD14 (a cell surface marker specific for monocyte/macrophage cell populations) were discarded. Seven-day-old MDMs were obtained, as previously described [13], by culturing monocytes for the first 3 days in RPMI 1640 supplemented with 20% heat-inactivated fetal calf serum (FCSi) (both from Lonza, Milan) and 50 ng/ml of granulocyte-macrophage colony-stimulating factor (a kind gift from Schering-Plough, Milan, Italy), to promote macrophage differentiation, and for the last 4 days in the same medium without granulocyte-macrophage colony-stimulating factor. THP-1 cell line, derived from an acute monocytic leukemia [58], was grown in RPMI 1640 supplemented with 10% FCSi. A549 cell line, derived from a human lung carcinoma [59], was grown in Dulbecco's modified minimal essential medium supplemented with 10% FCSi. Wild type myristoylated Nef_SF2_, a mutant in the acidic cluster (E^66^EEE^69^→AAAA), a mutant lacking the first 44 amino acids (N-term) and a myristoylation deficient mutant (G2A), were co-expressed with a N-myristoyl-transferase expression vector in *E.coli* induced in presence of myristic acid and purified as C-terminal hexahistidine-tagged fusion proteins as previously described [60]. Wild type as well as 4EA mutant preparations were checked by SDS-PAGE and Coomassie staining after purification (see Fig. S2). Nef preparations were analyzed for the presence of endotoxin using the chromogenic *Limulus* amebocyte lysate endpoint assay QCL-1000 and, if required, purified using the EndoTrap endotoxin removal system (both from Lonza). To avoid possible signalling effects due to residual lipopolysaccharide (LPS) traces in Nef preparations, all of the experiments were performed in the presence of 1 µg/ml of polymyxin B (Sigma-Aldrich, Milan, Italy), a cationic antibiotic that binds to the lipid A portion of bacterial LPS. In our hands, this polymyxin B treatment did not interfere with the signalling events analyzed and blocked the signalling activity of up to 50 endotoxin units (EU)/ml LPS.

### Circular Dichroism Spectroscopy

CD spectra were recorded on a Jasco J-815 spectropolarimeter at 25°C using a quartz cuvette with a path length of 1 mm. The myristoylated Nef proteins were dialyzed against 5 mM KP_i_ buffer (pH 8.2) and measured at a concentration of 5.0 µM. Spectra were recorded with a step size of 1 nm and an integration time of 1 s. The raw CD signal (in millidegrees), after subtraction of the blank signal of the cuvette and buffer, was converted to mean residue ellipticity (in degrees squared centimeters per decimole) similarly as described [59].

### Plasmids, constructs and GST fusion proteins

The plasmid expressing Nef acidic domain mutant, designated as pCDNA3-Nef 4EA, was obtained from vector expressing wild type Nef derived from NL4-3 Nef allele, by replacing Glu^62^ to Glu^65^ codons (corresponding to Glu^66^ to Glu^69^ in the SF2 allele) by alanines, using Stratagene QuickChange II site-directed mutagenesis kit (M-Medical, Milan, Italy) according to the manufacturer's instructions with minor modifications. In particular, we extended the PCR cycles to 25, we purifed PCR products after DpnI digestion using Wizard SV Gel and PCR Clean up system (Promega, Milan, Italy) and we transformed DH5 *E. coli* strain by electroporation with the purified PCR product. The following primers were used for the PCR reaction:

4EA Forward 5′-(p)cctggctagaagcacaagcggcggcagcggtgggttttcc-3′; 

4EA Reverse 5′-(p)ggaaaacccaccgctgccgccgcttgtgcttctagccagg-3′.

The mutant sequence was verified by DNA sequencing. Wild type Nef and mutated Nef 4EA were next introduced into pGEX6P3 (GE Healthcare, Milan, Italy) in two steps: initially the ORFs were subcloned into pET-28a(+) (Novagen/Merck, Darmstadt, Germany) using Hind III/Not I sites. Next, the ORF codifying Nef wt and Nef 4EA were excised from pET-28a(+) using the Bam HI site (5′ to Hind III), available in the pET-28a(+) multiple cloning region, and introduced into pGEX6P3 using Bam HI/Not I sites. Details of each construct are available upon request. All ligation reactions were performed using Quick Ligase (New England Biolabs, Ipswich, MA), according to the manufacturer's instructions.

### Western blot assay

MDMs were washed twice with phosphate-buffered saline (PBS), pH 7.4, and lysed in 20 mM HEPES, pH 7.9, 50 mM NaCl, 10 mM EDTA, 2 mM EGTA, 0.5% nonionic detergent IGEPAL CA-630 (Sigma-Aldrich), 0.5 mM dithiothreitol (DTT), 20 mM sodium molybdate, 10 mM sodium orthovanadate, 100 mM sodium fluoride, 10 µg/ml leupeptin, 0.5 mM phenylmethylsulfonyl fluoride for 20′ in ice. Whole-cell lysates were centrifuged at 6,000 *g* for 10′ at 4°C, and the supernatants were frozen at 80°C. The protein concentrations of cell extracts were determined by the Lowry protein assay. Aliquots of cell extracts containing 20 to 50 µg of total proteins were resolved on 7 to 10% sodium dodecyl sulfate-polyacrylamide gel electrophoresis (SDS-PAGE) and transferred by electroblotting them on nitrocellulose membranes (Sartorius, Gottingen, Germany) overnight at 30 V with a Bio-Rad Trans-Blot apparatus. For the immunoassays, membranes were blocked in 3% bovine serum albumin (BSA) fraction V (Sigma-Aldrich, Milan, Italy) in TTBS/EDTA (10 mM Tris, pH 7.4, 100 mM NaCl, 1 mM EDTA, 0.1% Tween 20) for 1 h at RT and then incubated for 1 h at RT or overnight at +4°C with specific antibodies diluted in 1% BSA/TTBS-EDTA. Antibodies used in the different immunoblottings were as follows: rabbit polyclonal antibodies anti-phospho-IKKα(Ser180)/IKKβ(Ser181), anti-IKKα, anti-IKKβ, anti-IκB-α, anti-phospho-Stat1(Tyr701), anti-phospho-Stat3(Tyr705), all from Cell Signaling Technology (Beverly, MA); rabbit polyclonal antibodies anti-STAT1 (E-23), anti-STAT2 (C-20), anti-STAT3 (C-20), anti-IRF-3 (FL-425), anti-Hck (M-28), anti-TRAF2 (C-20) and anti-TRAF6 (H-274) all from Santa Cruz Biotechnology (Santa Cruz, CA); mouse monoclonal anti β-tubulin from ICN Biomedicals (Costa Mesa, CA); rabbit polyclonal anti-phospho-STAT2(Tyr689) from Upstate Biotechnology/Millipore (Billerica, MA). Immune complexes were detected with horseradish peroxidise conjugated goat anti-rabbit or goat anti-mouse antiserum (Calbiochem), followed by enhanced chemiluminescence reaction (ECL; Amersham Pharmacia Biotech, Milan, Italy). To reprobe membranes with antibodies having different specificities, nitrocellulose membranes were stripped for 5′ at RT with Restore Western Blot Stripping Buffer (Pierce, Rockford, IL) and then extensively washed with TTBS/EDTA.

### Pull-Down Assays

GST-Hck_SH3_ pull down assay with Nef protein were prepared in 10 mM Tris buffer, pH 8.0, and subjected to SDS PAGE analysis, similarly as described [58]. For TRAF2/GST-Nef pull down assay, GST-Nef wt or GST-Nef 4EA fusion proteins were expressed and purified according to the described procedure [61]. For pull down experiments, THP-1 cells were lysed in pull-down buffer (20 mM Hepes pH 7.5, 50 mM NaCl, 10% Glicerol, 10 mM EDTA, 2 mM EGTA, 0,5% Igepal CA-630, 1 mM Na_3_VO4, 10 mM Na_2_MoO4, 0,5 mM PMSF, 2 mg/ml Aprotinin, 2 mg/ml Leupeptin, all from Sigma-Aldrich). Next 40 µg of GST-fusion proteins immobilized onto GSH beads were incubated with 2 mg of total cellular lysate for 3 hours at 4°C in a pull-down buffer. Specifically bound proteins were resolved on an SDS-PAGE (9%) and transferred overnight onto nitrocellulose membranes. GST and GST-Nef were detected by Ponceau S staining. Membranes were then blocked in TBS buffer containing BSA (3%) for 1 h and then incubated overnight with anti-TRAF2 or anti-Hck antibodies, followed by detection with horseradish peroxidase-conjugated anti-rabbit IgG and the ECL.

### RNA isolation and real-time PCR

Real-time PCR assays were performed on total RNA isolated from MDMs treated or not for 2 h with 100 ng/ml of recombinant Nef or its mutants, using the RNeasy mini kit (Qiagen, Milan) according to the manufacturer's instructions. Five hundred nanograms of total RNA was reverse transcribed using oligo(dT)12–18 (Pharmacia-Biotech) as a primer and 50 units of Moloney murine leukemia virus reverse transcriptase enzyme (Gibco/Invitrogen, Milan). Quantitative real-time PCR was then performed on reverse-transcribed IFNβ, IL-1β, IL-6, TNF-α, CCL3/MIP-1α and CCL4/MIP-1β mRNAs. The expression of GAPDH (glyceraldehyde-3-phosphate dehydrogenase) was used to normalize the mRNAs levels. Basal mRNA level observed in untreated cells, was chosen as a calibrator. Oligonucleotides used in the RT-PCR are reported in Table 2.

**Table 2:** Oligonucleotides used to perform RT-PCR.

Gene (Accession Number*)	Oligonucleotide Sequence	Amplicon lenght (bp)	Refs.
IFNβ (NM_002176.2)	Fwd: 5′-CAGCAGTTCCAGAAGGAGGA-3′	105	[13]
	Rev: 5′-AGTCTCATTCCAGCCAGTGC-3′		
IL-1β (NM_000576.2)	Fwd: 5′-GCTTATTACAGTGGCAATGAGGAT-3′	130	[62]
	Rev: 5′-GGTGGTCGGAGATTCGTAGC-3′		
IL-6 (NM_000600.3)	Fwd: 5′-GGTACATCCTCGACGGCATCT-3′	81	[63]
	Rev: 5′-GTGCCTCTTTGCTGCTTTCAC-3′		
TNF- (NM_000594.2)	Fwd: 5′-ATCTTCTCGAACCCCGAGTGA-3′	83	[64]
	Rev: 5′-CGGTTCAGCCACTGGAGCT-3′		
CCL3/MIP-1 (NM_002983.2)	Fwd: 5′-GAGACGAGCAGCCAGTGCTC-3′	56	
	Rev: 5′-CGGCTTCGCTTGGTTAGGA-3′		
CCL4/MIP-1β (NM_002984.2)	Fwd: 5′-CCATGAAGCTCTGCGTGACT-3′	82	[62]
	Rev: 5′-AGCCCATTGGTGCTGAGAG-3′		
GAPDH (NM_002046.3)	Fwd: 5′-GGGAAGGTGAAGGTCGGAGT-3′	101	[13]
	Rev: 5′-TCATTGATGGCAACAATATCCACT-3′		

*Accession number according to NCBI Reference Sequence.

### Antiviral assay

The antiviral activities of supernatants collected from Nef-treated MDMs were tested on A549 cells. A 96-well microtiter plate (Greiner bio-one, Frickenhausen, Germany) was seeded with 4×10^4^ A549 cells/well in Dulbecco's modified minimal essential medium, 2% FCSi. After 24 h, the culture medium was replaced with twofold dilutions of Nef-treated MDM conditioned medium (100 µL). Twenty-four hours later, cell cultures were infected with 100 µl of a 1∶1,000 dilution of murine encephalomyocarditis virus (EMC viral titer: 2.8×10^8^ PFU/ml) and incubated for an additional 24 to 48 h. The cytopathic effect was visualized under inverted microscopy, and antiviral activity was calculated using a preparation of recombinant IFNβ (Rebif; 9×10^7^ IU/ml, 3×10^8^ IU/mg of protein; Ares-Serono) as a standard.

### Nef labelling, cytofluorimetric (CFM) assay and confocal microscopy (CM)

One hundred micrograms of wild type and 4EA myr^+^ recNef_SF2_ was labelled using AlexaFluor488 Microscale Protein Labelling Kit (Molecular Probes/Invitrogen, Italy) following the manufacturer's recommendations. The labelled protein concentration and the degree of labelling (DOL) were determined by spectrophotometric methods. The DOL of wild type and 4EA myr^+^ recNef_SF2_-Alexa-488 conjugated preparations ranging from 2.42 and 2.68 moles dye/mole protein and the conjugated protein concentrations were 275 and 153 µg/ml, respectively. For CFM analysis, cells were treated with myr^+^ recNef_SF2_-Alexa488 (300 ng/ml), then washed twice with phosphate-buffered saline pH 7.4 (PBS) 2% FBS, harvested and fixed in PBS 2% paraformaldehyde for 60′ on ice. Afterwards, cells were washed once with PBS and CFM analysis was performed using Galaxy flow cytometer (laser 488 m, 25 mW, on FL1 channel, pass-band filter to 520 nm). Fluorescence analyses were performed using the FLOMAX- v.2.4b software. For CM, MDMs were cultured on glass bottom dishes (MatTek Corporation, Ashland, MA) and treated for 30′ with 300 ng/ml wt or 4EA myr^+^ recNef_SF2_-Alexa488, then fixed in PBS 2% paraformaldehyde for 60′ on ice, washed four times with PBS and analysed using confocal microscope (Leica TCS SP5). Plasma membrane counterstaining was performed by treating MDMs for 5′ with PKH26-GL using PKH26 Red Fluorescent Cell Linker Kit for General Cell Membrane Labeling (Sigma-Aldrich) following the manufacturer's recommendations before fixing the cells.

### THP-1 transfection, TRAF2 and TRAF6 silencing

Silencing experiments were performed using TRAF2 and TRAF6 specific SMARTpool siRNA and DharmaFECT 2 reagent (both from Dharmacon/Thermo Fisher Scientific, Lafayette, CO). As negative control a HPV16 E7 specific siRNA was used. In particular THP-1 cells were resuspended in Optimem reduced-serum medium (Gibco/Invitrogen) and seeded at 2×10^5^ cells/ml, 1 ml per well in a 6 well plate. At the same time both siRNA (1 µM) and DharmaFECT (1∶100 dilution) were separately incubated at room temperature for 5′ in Optimem, then mixed together in a 1∶1 ratio and incubated at room temperature for additional 5′. One hundred microlitres of this mixture was added to each well (final concentration of the siRNA: 100 nM). Twenty four hours later 1 ml of Optimem was added to each well. After additional 24 h wells were supplemented with 2 ml of Optimem, 20% FCSi, 2,4% Dimetilsulfoxide (Sigma-Aldrich). Seventy two hours post transfection, cells were collected, washed 1× in PBS, resuspended in RPMI 1640, 10% FCSi and incubated for 6 h with 200 ng/ml recNef_SF2_.

## Supporting Information

Figure S1 **Nef-induced upregulation of both TNF and IL-6 mRNAs depend on NF-κB pathway.** Primary human MDMs were pretreated for 1 h at 37°C with the highly selective IKK/IKKβ, inhibitor BMS-345541 (25 µM), then incubated for 2 h at 37°C with myr^+^ wild type recNef. Cells were processed and RT-PCR performed as described in the correspondent Materials & Methods section. Primers used for RT-PCR were listed in Table II.
(TIF)

Figure S2 **Coomassie gel showing both myr^+^ recNef WT and 4EA proteins used to stimulate cells.** Two µg of myr^+^ recNef variants were loaded on 18% SDS-PAGE. Gel was stained using Coomassie dye. Arrows indicate wild type and 4EA myr^+^ recNef.
(TIF)
