# Supplementary figures and images for: HIV-1 Nef Induces Proinflammatory State in Macrophages through Its Acidic Cluster Domain: Involvement of TNF Alpha Receptor Associated Factor 2
Source: PLoS One. 2011 Aug 23;6(8):e22982. doi: 10.1371/journal.pone.0022982 (PMC3160284; doi:10.1371/journal.pone.0022982)

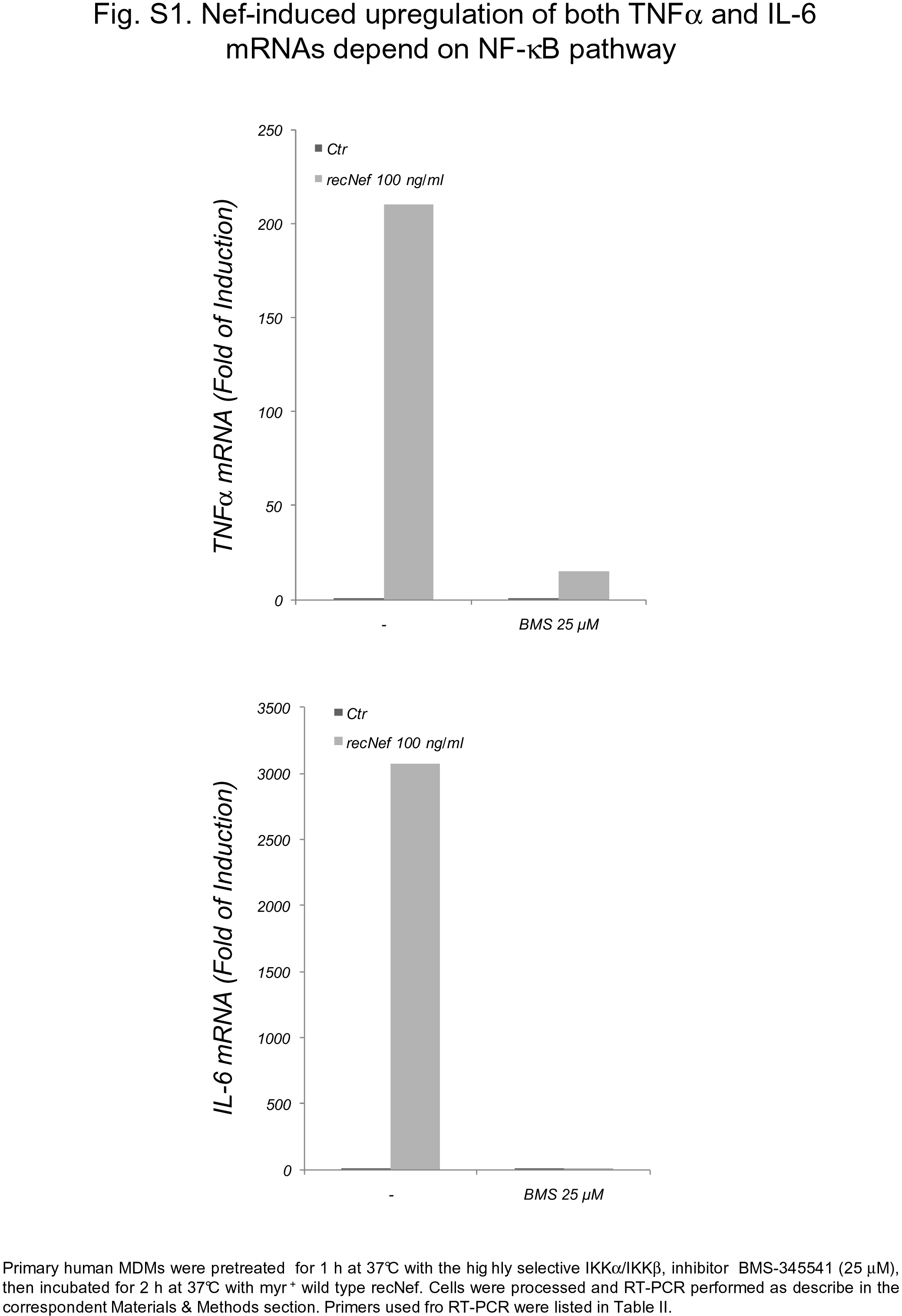

Supplement: Figure S1 — Nef-induced upregulation of both TNF and IL-6 mRNAs depend on NF-κB pathway. Primary human MDMs were pretreated for 1 h at 37°C with the highly selective IKK/IKKβ, inhibitor BMS-345541 (25 µM), then incubated for 2 h at 37°C with myr+ wild type recNef. Cells were processed and RT-PCR performed as described in the correspondent Materials & Methods section. Primers used for RT-PCR were listed in Table II. (TIF) [file pone.0022982.s001.tif]

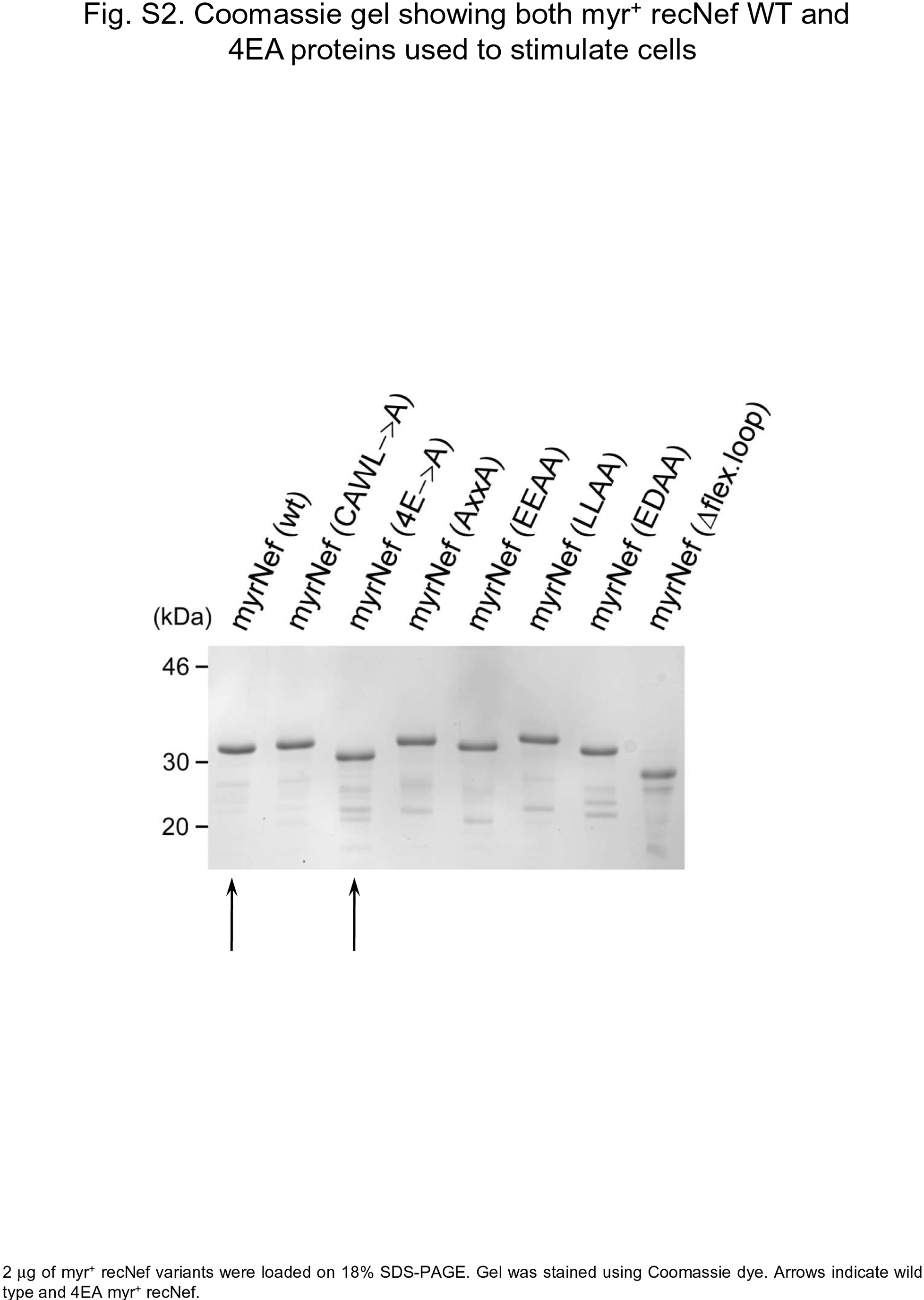

Supplement: Figure S2 — Coomassie gel showing both myr+ recNef WT and 4EA proteins used to stimulate cells. Two µg of myr+ recNef variants were loaded on 18% SDS-PAGE. Gel was stained using Coomassie dye. Arrows indicate wild type and 4EA myr+ recNef. (TIF) [file pone.0022982.s002.tif]
